# CsPbBr_3_ and Cs_2_AgBiBr_6_ Composite Thick Films with Potential Photodetector Applications

**DOI:** 10.3390/ma17205123

**Published:** 2024-10-21

**Authors:** Merida Sotelo-Lerma, Leunam Fernandez-Izquierdo, Martin A. Ruiz-Molina, Igor Borges-Doren, Ross Haroldson, Manuel Quevedo-Lopez

**Affiliations:** 1 Department of Research in Polymers and Materials, Universidad de Sonora, Blvd. Luis Encinas y Rosales S/N, Hermosillo 83000, Sonora, Mexico; merida.sotelo@unison.mx (M.S.-L.); a214206115@unison.mx (M.A.R.-M.); a218230145@unison.mx (I.B.-D.); 2 Materials Science and Engineering Department, University of Texas at Dallas, 2601 North Floyd Road, RL 10, Richardson, TX 75080, USA; lxf180007@utdallas.edu (L.F.-I.); reh101020@utdallas.edu (R.H.)

**Keywords:** perovskite tick film, composite, PBM, photodetector

## Abstract

This paper investigates the optoelectronic properties of CsPbBr_3_, a lead-based perovskite, and Cs_2_AgBiBr_6_, a lead-free double perovskite, in composite thick films synthesized using mechanochemical and hot press methods, with poly(butyl methacrylate) as the matrix. Comprehensive characterization was conducted, including X-ray diffraction (XRD), Raman spectroscopy, scanning electron microscopy (SEM), UV–visible spectroscopy (UV–Vis), and photoluminescence (PL). Results indicate that the polymer matrix does not significantly impact the crystalline structure of the perovskites but has a direct impact on the grain size and surface area, enhancing the interfacial charge transfer of the composites. Optical characterization indicates minimal changes in bandgap energies across all different phases, with CsPbBr_3_ exhibiting higher photocurrent than Cs_2_AgBiBr_6_. This is attributed to the CsPbBr_3_ superior charge carrier mobility. Both composites showed photoconductive behavior, with Cs_2_AgBiBr_6_ also demonstrating higher-energy (X-ray) photon detection. These findings highlight the potential of both materials for advanced photodetector applications, with Cs_2_AgBiBr_6_ offering an environmentally Pb-free alternative.

## 1. Introduction

Photodetectors play a fundamental role in a wide range of technologies, including optical communication systems, imaging devices, and biomedical sensing applications. Photodetectors convert incoming light into electrical signals, and most devices are generally categorized into photodiodes and photoconductors, with the latter standing out for their simpler metal–semiconductor–metal structures and capacity to generate high photocurrents [1,2,3]. The relatively simple device geometry makes photoconductors an attractive choice for planar device designs [4,5]. The key performance metrics of these devices, including responsivity, sensitivity, and wavelength selectivity, are primarily determined by the properties of the semiconductor material used [6].

Metal halide perovskites, particularly those following the general formula ABX_3_, have gained widespread attention due to their remarkable optical and electronic properties. CsPbBr_3_, a lead-based perovskite, is particularly interesting due to its exceptional photoresponse and carrier transport capabilities, making it a highly promising candidate for photodetector applications. However, concerns regarding the toxicity and environmental impact of lead have prompted the exploration of alternative lead-free materials [7,8,9]. Further, CsPbBr_3_ suffers from limited long-term stability.

In this context, Cs_2_AgBiBr_6_, a lead-free double perovskite, is an interesting Pb-free potential solution owing to its stable crystal structure, non-toxic nature, and promising optoelectronic characteristics [10,11,12]. Despite these advantages, several challenges remain for Cs_2_AgBiBr_6_. Although Cs_2_AgBiBr_6_ is more stable, further optimization is needed to achieve comparable efficiency to lead-based perovskite systems. Additionally, scalable synthesis methods are still required for both CsPbBr_3_ and Cs_2_AgBiBr_6_ [13].

One promising fabrication technique for these perovskites is mechanochemical synthesis. Mechanochemical methods involve the grinding of precursor materials with the desired stoichiometric ratios in a solvent-free environment. This is a scalable approach that has been demonstrated to produce high-quality perovskite materials, including CsPbBr_3_ and Cs_2_AgBiBr_6_ [14,15]. Composites of these materials can be prepared once synthesized in powder form using mechanochemical methods by using polymers as the matrix. This enables a straightforward route to integrate these materials into large-scale, cost-effective photodetector systems.

In this study, the optoelectronic properties of composite thick films of CsPbBr_3_ and Cs_2_AgBiBr_6_ synthesized via mechanochemical and hot press methods are investigated using comprehensive structural, optical, and electronic characterization using X-ray diffraction (XRD, Rigaku, Smartlab, Cedar Park, TX, USA), Raman spectroscopy (ThermoFisher Scientific, Microscope DXR, Waltham, MA, USA), scanning electron microscopy (SEM, Zeiss, Supra 40, USA), UV–visible spectroscopy (UV–Vis, Agilent Technologies, Cary Series, Santa Clara, CA, USA), and photoluminescence (PL). The results demonstrate the potential of lead-based and lead-free perovskites for next-generation photodetectors and offers insights into their stability, performance, and scalability.

## 2. Materials and Methods

Cesium bromide (CsBr, ultradry, 99.9%) and silver(I) bromide (AgBr, 99.5%) were acquired from Alfa-Aesar (Fort Worth, TX, USA). Lead(II) bromide (PbBr_2_, 98+%) and bismuth(III) bromide (BiBr_3_, 99%) were acquired from Thermoscientific (Houston, TX, USA). Poly(butyl methacrylate) (PBM, MW 200,000 g/mol) was acquired from Aldrich (Austin, TX, USA). The stainless-steel balls (3 mm), stainless-steel milling jars, and the high-energy vertical planetary ball mill were acquired from MSE Supplies LLC (Tucson, AZ, USA).

### 2.1. Preparation of the Perovskite Powder Precursors

CsPbBr_3_ synthesis: stoichiometric amounts, 2.5 mmol of CsBr and 2.5 mmol of PbBr_2_, were added into the jars of a planetary ball milling containing 5 g of stainless-steel balls with a diameter of 3 mm per gram of precursors. The jars were then placed in the vertical planetary ball-milling system for a total solid-state reaction time of 12 h at a constant rotational velocity of 700 rpm and at room temperature. Preliminary results suggest that the order in which the precursors and balls are added to the jar plays an essential role and indicate that higher quality materials are obtained when the precursors are added before the balls. The same methodology was followed for all materials prepared [14].

Cs_2_AgBiBr_6_ synthesis: stoichiometric amounts were used for the mechanochemical synthesis by planetary ball milling: 5.0 mmol of CsBr, 2.5 mmol of BiBr_3_, and 2.5 mmol AgBr, with a 2:1:1 mol ratio, and 5 g of stainless-steel balls with a diameter of 3 mm per gram of precursors was first added to the jars. This process was followed for all experiments described in this study, with a solid-state reaction time of 12 h at a constant rotational velocity of 700 rpm and at room temperature.

### 2.2. Preparation of the Thick Composite Films

**Figure 1:**
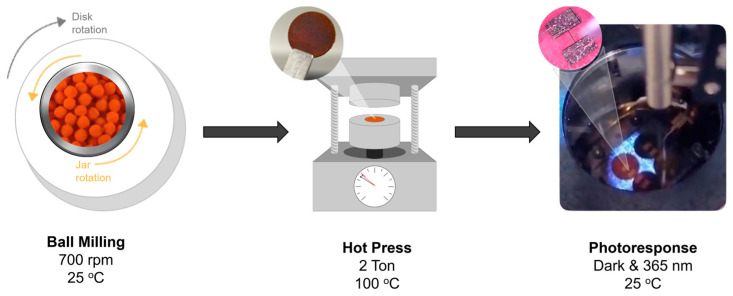
Flow chart of device preparation for halide perovskite composites. Ball milling was used for the mechanochemical synthesis of perovskite materials. The hot press was used to obtain the thick film using a composite (perovskite:polymer—75:25 wt%). The photoresponse under dark and illuminated conditions (photons of 365 nm) was used to characterize the device.

Polymer composites of CsPbBr_3_ and Cs_2_AgBiBr_6_: a physical mixture of CsPbBr_3_–PBM and Cs_2_AgBiBr_6_–PBM was prepared by simply mixing the powders following manual grinding at a 75:25 weight percentage. PBM is a poly(butyl methacrylate) with a molecular weight of 200,000 g/mol. A total of 200 mg of the obtained powder was compressed with 2 tons of force and heated at 100 °C for 30 min with a Carver standard pellet press connected to the WK-1 heating system (Figure 1). The expected thickness was between 400–700 µm, so that the thick film could be self-supporting and present good mechanical properties.

### 2.3. Characterization

To identify the nature of the crystalline phases, X-ray diffraction (XRD) was carried out in a Rigaku SmartLab with a Cu X-ray anode (Kα1 = 0.15406 nm) using a Bragg–Brentano geometry. Ultraviolet–visible spectroscopy was performed in an Agilent Technologies Cary Series UV-Vis-NIR spectrophotometer, and morphology for the precursor materials and thick composite films were characterized by a scanning electron microscope (SEM) using a SEM ZEISS SUPRA 40. The Raman spectra of the powders and the thick films were obtained using a Raman spectrometer Thermo scientific Microscope DXR with a 532 nm wavelength excitation source.

## 3. Results

**Figure 2:**
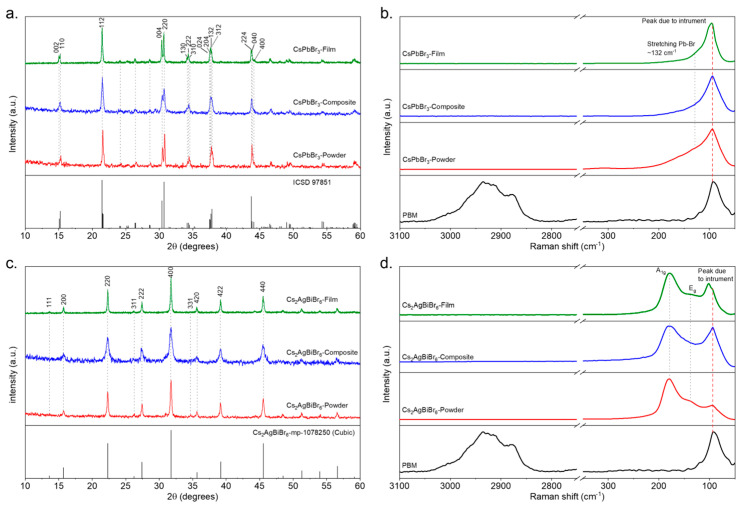
XRD diffractograms of (**a**) CsPbBr_3_ and (**c**) Cs_2_AgBiBr_6_. Raman spectra with 780 nm laser source of (**b**) CsPbBr_3_ and (**d**) Cs_2_AgBiBr_6_.

The XRD patterns for the CsPbBr_3_ powders, as shown in Figure 2a, exhibited prominent diffraction peaks at 15.20°, 21.46°, and 30.66°, corresponding to the (110), (112), and (220) planes, respectively. These peaks are characteristic of the orthorhombic crystal structure of the Pbnm special group and of lattice parameters obtained using the Vesta software (VESTA, version 3.5.8, built on 11 August 2022) via the ICSD 97851 reference file and are consistent with previous reports [16,17,18,19]. These parameters included a = 8.207 Å, b = 8.255 Å, c = 11.70 Å and their indexing using the raw data, i.e., a = 8.217 Å, b = 8.298 Å, c = 11.757 Å. The XRD patterns for the Cs_2_AgBiBr_6_ powders, displayed in Figure 2c, revealed peaks at 15.71°, 22.31°, 27.41°, and 31.75°, corresponding to the (200), (220), (222), and (400) planes, respectively. This is indicative of a cubic structure [20,21] of the space group Fm$\bar{3\text{m}}$ and of lattice parameters obtained using the Vesta software via the reference file mp-1078250, namely a = 11. 255 Å and its indexing using the raw data, i.e., a = 11.258 Å.

**Figure 3:**
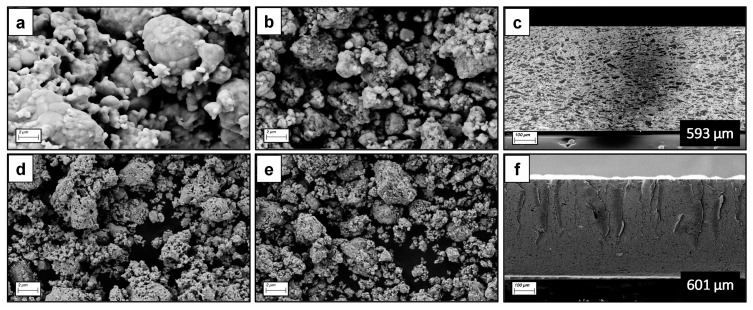
SEM images for (**a**) CsPbBr_3_ (powder), (**b**) CsPbBr_3_ (composite), (**d**) Cs_2_AgBiBr_6_ (powder), (**e**) Cs_2_AgBiBr_6_ (composite), and cross-section images for (**c**) CsPbBr_3_ (film) and (**f**) Cs_2_AgBiBr_6_ (film).

The XRD patterns for the CsPbBr_3_ and Cs_2_AgBiBr_6_ thick composite films are shown in Figure 2a,c. Results indicate that the polymer matrix did not alter the fundamental crystal structures of the perovskites. However, a noticeable broadening of the diffraction peaks was observed in the composites. This peak broadening suggests a reduction in the perovskite grain size, which is attributed to the polymer matrix separating the perovskite grains, thus limiting grain growth, while simultaneously holding them together, as depicted in Figure 3. The reduction in grain size likely contributed to the increased surface area and, potentially, enhanced the interfacial charge transfer within the composite. Interestingly, the crystalline structure of the perovskites remained intact even after subjecting the systems to pressure and temperature for the thick film fabrication.

The compositional and structural integrity of the resulting materials was further investigated using Raman spectroscopy. The CsPbBr_3_ perovskite exhibited distinct vibrational modes at 72–77 cm^−1^, attributed to [PbBr_6_]^4−^ octahedral vibrations, and at 132 cm^−1^, corresponding to the transverse optical phonon of the Pb–Br stretching mode [22,23,24]. However, in the Raman spectrum shown in Figure 2b, a dominant peak at ~100 cm^−1^, attributed to the measurement instrument, complicated the observation of the characteristic perovskite vibrational modes. For Cs_2_AgBiBr_6_, the Raman spectrum (Figure 2d) showed characteristic peaks at 178.6 cm^−1^ and 138.14 cm^−1^, associated with the Ag–Br and Bi–Br bond vibrations within the [AgBr_6_]^5−^ and [BiBr_6_]^3−^ octahedra, respectively. These modes were assigned to the A_1g_ and E_g_ vibrational modes, consistently with previous reports [25,26,27]. The polymer’s Raman signature was observed in the 2800–3100 cm^−1^ region, corresponding to C–H stretching vibrations [27]. Importantly, as seen in Figure 2b,d, no significant shifts or changes in the Raman spectra of the perovskite–polymer composites were detected, suggesting that the perovskite structure remained stable upon interaction with the polymer matrix. However, they exhibited an increase in peak broadening and their ratio, possibly due to the dilution of the active material (perovskite). The polymer may have coated the perovskite particles, which may have led to the reduction of the direct interaction between the Raman laser and the perovskite surface, affecting the intensity of the peaks. On the other hand, the peak around 138.14 cm^−1^ was classified as E_g_ due to the asymmetric stretching vibrational modes of Ag–Br and Bi–Br bonds appearing to have a lower intensity than Ag around 178.6 cm^−1^, corresponding to the symmetric stretching vibrational modes; the polymer coating could have acted as a barrier, limiting the vibrational response of the bonds within the perovskite.

### 3.1. Powers and Composite Morphology

The grain size of CsPbBr_3_ and Cs_2_AgBiBr_6_ perovskites, both as powders and as composites, was affected by the interaction with the polymer matrix and the porosity generated in the composite films. The incorporation of the polymer resulted in the intergrain separation of the perovskites, as depicted in Figure 3b,e. The polymer functions as a host matrix, not only dispersing the perovskite grains but also enhancing the composite’s mechanical properties by providing structural support and flexibility [28].

As observed in the cross-sectional images (Figure 3c,f), the polymer integration led to highly compact and homogeneous composite films. The polymer matrix reduced the number of voids and the amount of porosity, a phenomenon which is crucial for ensuring efficient charge transport and for improving the overall performance of the composite in potential photodetector applications. The thickness of the resulting composite films was 593 µm for the CsPbBr_3_-based film and 601 µm for the Cs_2_AgBiBr_6_-based film. These uniform thicknesses indicate a consistent hot-pressing process, which is essential for maintaining the optical and electronic properties across the entire film area. Dense films and precise control over thickness are critical factors that can directly influence the sensitivity and performance of the films in optoelectronic devices.

In summary, the incorporation of the polymer matrix into the perovskite composites led to well-dispersed grain structures, reduced porosity, and improved mechanical stability of the films. These characteristics contribute to the potential of CsPbBr_3_ and Cs_2_AgBiBr_6_ composite films as high-performance photodetectors, where uniformity and structural integrity are key to enhance device functionality.

### 3.2. Optical Properties of the Composite Films

The optical properties of the CsPbBr_3_ and Cs_2_AgBiBr_6_ perovskites were investigated using UV–Vis diffuse reflectance spectroscopy and photoluminescence (PL) at room temperature. The bandgap energy (E_g_) was determined using the Kubelka–Munk theory applied to the diffuse reflectance data.

**Figure 4:**
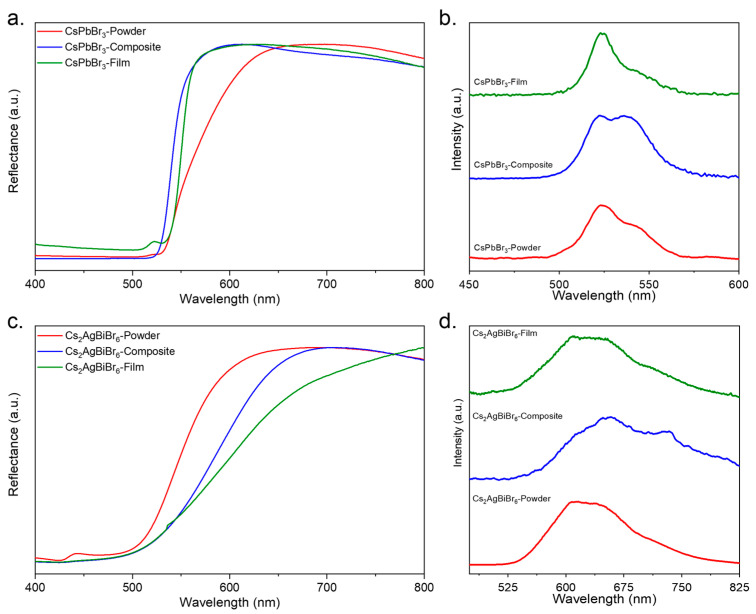
UV–Vis spectroscopy of (**a**) CsPbBr_3_ and (**c**) Cs_2_AgBiBr_6_. PL spectroscopy of (**b**) CsPbBr_3_ and (**d**) Cs_2_AgBiBr_6_.

For the CsPbBr_3_ perovskite, as shown in Figure 4a, the diffuse reflectance spectra of the powder exhibited a decay starting at 650 nm, while both the composite and the film showed a blue shift, with the onset of decay occurring at 580 nm. This shift was attributed to the presence of the polymer in both the composite and the film, separating the perovskite grains and leading to changes in the material’s optical properties. The powder, lacking the polymer matrix, maintained a closer grain proximity, which resulted in an orange hue under visible radiation. In contrast, the composite displayed a slightly lighter shade of orange, and, upon transformation into a film, the color reverted to that of the original powder. The calculated E_g_ values for the powder, composite, and film were 2.29 eV, 2.34 eV, and 2.29 eV, respectively, indicating a slight increase in bandgap energy due to polymer interaction in the composite [29,30,31,32].

The photoluminescence spectrum of CsPbBr_3_, shown in Figure 4b, revealed two prominent peaks at 541 nm and 524 nm. The peak at 541 nm (2.29 eV) corresponded to the direct recombination of free excitons, consistent with the bandgap energy. The peak at 524 nm (2.37 eV) was attributed to defect states caused by Br vacancies in the perovskite lattice [6,30,33]. This dual peak behavior indicates the presence of both intrinsic excitonic emissions and defect-mediated emissions.

For Cs_2_AgBiBr_6_, the UV–Vis diffuse reflectance spectra (Figure 4c) showed a red shift in the onset of reflectance decay compared to CsPbBr_3_. The powder exhibited a decay starting at 672 nm, while the composite and the film exhibited a further shift, with decay beginning at 695 nm. The calculated indirect bandgap energies for Cs_2_AgBiBr_6_ were 2.26 eV, 2.24 eV, and 2.22 eV for the powder, composite, and film, respectively, indicating a slight narrowing of the bandgap upon polymer incorporation [34,35].

The photoluminescence spectrum of Cs_2_AgBiBr_6_, shown in Figure 4d, showed a broad peak with two distinct contributions. The peak at approximately 610 nm corresponded to the material’s indirect bandgap (2.26 eV), while the second peak at around 700 nm can be attributed to two possible mechanisms. The first is photon-assisted indirect band transitions, where the transition requires assistance from phonons to conserve momentum [35]. The second mechanism, as suggested by Ping Fan et al., is a direct bandgap emission from Cs_2_AgBiBr_6_ [36].

The evaluation of E_g_ for the perovskite materials in powder, composite, and film forms indicated that the optical bandgap remained stable across different phases. Notably, the E_g_ values for the films closely matched those of the pristine powders, suggesting minimal alteration of the fundamental electronic structure upon interaction with the polymer. Moreover, the photoluminescence spectra for both materials maintained the characteristic peaks observed in the powder form, with no additional peaks arising from polymer interactions, as you can see in Figure S1. This stability in both the optical bandgap and PL behavior highlights the robustness of CsPbBr_3_ and Cs_2_AgBiBr_6_ when incorporated into polymer matrices, making them promising candidates for optoelectronic applications, particularly in photodetectors.

### 3.3. Electrical Characterization

**Figure 5:**
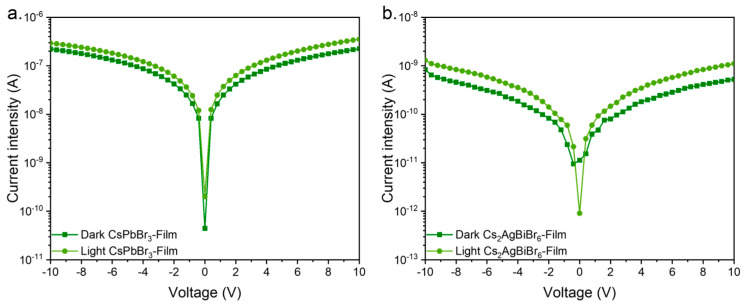
I–V curves of (**a**) CsPbBr_3_ and (**b**) Cs_2_AgBiBr_6_.

Devices were fabricated using 200 nm gold contacts to evaluate the current–voltage (I–V) characteristics of the devices in total darkness and under illumination using a 365 nm LED light source. The applied voltage sweep ranged from −10 V to 10 V. The resulting I–V curves are shown in Figure 5. As expected, both CsPbBr_3_ and Cs_2_AgBiBr_6_ films exhibited a significant increase in photocurrent when exposed to illumination compared to photocurrents taken in the dark. This behavior indicates the photoresponsive nature of both materials, confirming their potential as photodetector materials. The increase in photocurrent can be attributed to the generation of electron–hole pairs upon exposure to light, which reduces the overall resistivity of the films and enhances the charge transport through the device.

Interestingly, the photocurrent values of the CsPbBr_3_-based devices were consistently higher than those observed for the Cs_2_AgBiBr_6_-based devices. This is possibly linked to the intrinsic properties of the CsPbBr_3_ perovskite, such as its higher absorption coefficient and more efficient charge carrier mobility, both of which contribute to improved photogenerated charge separation and transport.

Moreover, the decrease in resistivity under illumination suggests that both materials exhibit photoconductive properties, with the increased charge carrier density upon light exposure leading to enhanced electrical conductivity. This characteristic is particularly desirable for photodetector applications, as it ensures that the material can effectively respond to incident light with a rapid change in current.

**Figure 6:**
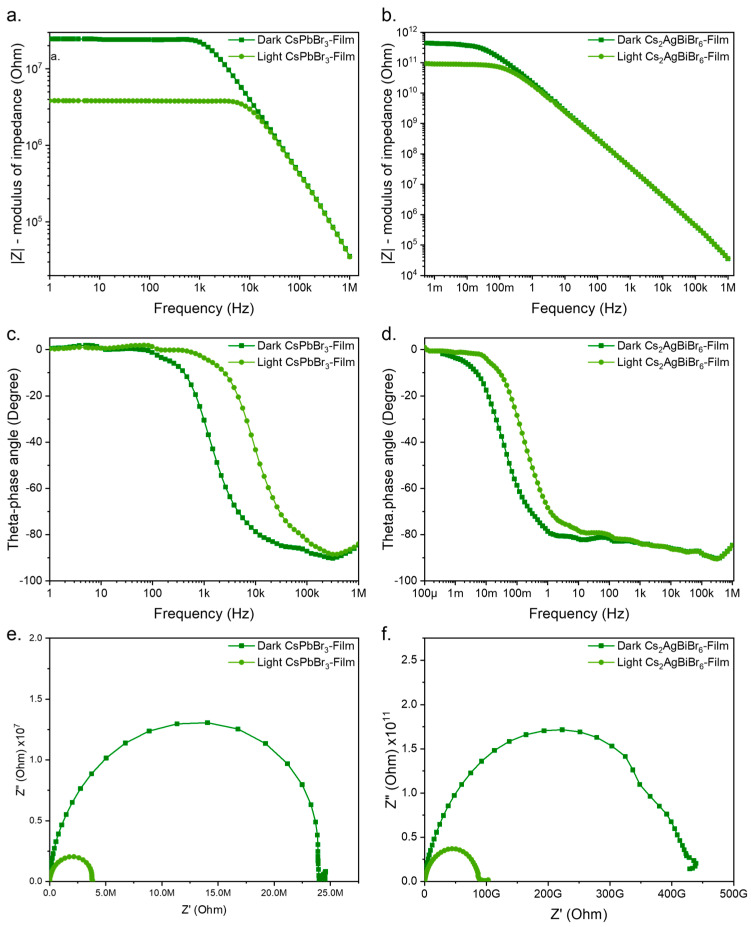
Bode diagram of (**a**) CsPbBr_3_ and (**b**) Cs_2_AgBiBr_6_. Theta phase vs. frequency of (**c**) CsPbBr_3_ and (**d**) Cs_2_AgBiBr_6_. Nyquist plot of (**e**) CsPbBr_3_ and (**f**) Cs_2_AgBiBr_6_.

The impedance spectra for the CsPbBr_3_ films as a function of frequency under both dark and illuminated conditions are presented in Figure 6e. A significant 20.57 MΩ difference was observed at low frequencies between the measurements in the dark and those under illumination. The overall resistance of the CsPbBr_3_-based composite was much higher than what has been reported for other perovskites with similar compositions [37]. This increase in resistance can be attributed to the incorporation of the polymer matrix, which influences the electrical properties by creating intergrain separation. Additionally, the small series resistance is reflected in the minimal shift to the right of the semicircles in the Nyquist plots shown in Figure 6e.

To model the electrical behavior of the devices, equivalent circuits were constructed using data from the electrochemical impedance spectroscopy (EIS) measurements, under both dark and illuminated conditions. These circuits, which can be found in Figure S2, were simulated using the Gamry Echem Analyst software (version 7.8.5). The circuit for the device in the dark consisted of several key elements: *R_s_* represents the resistance between the contacts and the film surface, *R_tr_* and *R_ct_* represent the transport and charge transfer resistances within the material, respectively, while *C_g_* and *C_d_* account for the geometric and dielectric capacitance, respectively. The circuit also included an inductance component *L_s_*, associated with the measuring cables [38]. A bounded Warburg element was employed to model diffusion effects and improve fitting.

Under dark conditions, the sum of *R_tr_* and *R_ct_* gave the total resistance, which corresponded to the real part of the impedance (24.3 MΩ) seen in the Nyquist plot for this device. Under illumination, the circuit elements remained largely the same, except for *R_ct_* and *C_d_* which represent the resistances and capacitances, respectively, of the material near the surface. These parameters decreased significantly when the film was exposed to light, accounting for the reduction in impedance. The total resistance under illumination, *R_p_*, matched the value observed in the Nyquist diagram (Figure 6e).

The changes in capacitance and diffusion function coefficients between dark and illuminated conditions also provide insights into the material’s properties. Parameters such as the dielectric constant and charge mobility underwent significant changes following illumination. These variations are captured in the phase diagrams [39] presented in Figure 6c,d. While these results highlight changes in complex impedance, our focus in this work is on the real impedance values, which support the earlier findings of increased photocurrent under illumination. The application of Ohm’s Law confirmed that a reduction in resistance leads to the increase in photocurrent observed in the I–V measurements.

Similar trends were observed with respect to the Cs_2_AgBiBr_6_ films, where the resistance decreased under illumination compared to the dark condition. However, the films exhibited a much higher overall resistance, as shown in the Nyquist diagram (Figure 6f). The real part of the impedance under dark conditions reached an extraordinarily high value of 424 GΩ, which decreased to 87 GΩ under illuminated conditions. These values were significantly greater than those recorded for the CsPbBr_3_-based devices, suggesting that the Cs_2_AgBiBr_6_ composite, which includes a double-cation perovskite and a polymer, possesses substantially higher resistivity.

To further investigate this behavior, we constructed and simulated equivalent circuits for the dark and illuminated conditions of the Cs_2_AgBiBr_6_ films. The components in these circuits were similar to those used for CsPbBr_3_. The main differences were the values of the resistive elements. Under illuminated conditions, the total resistance of the device decreased by nearly four times compared to the dark condition. Despite this, the Cs_2_AgBiBr_6_ material remained more resistive than CsPbBr_3_, even under illumination.

This higher resistivity is a crucial finding and suggests that the Cs_2_AgBiBr_6_ composite could be more suited for applications involving the detection of higher-energy photons, such as X-rays [9]. Materials with a high resistivity are often preferred for X-ray detectors because they reduce noise from eddy currents and are capable of being polarized at high voltages [40]. Moreover, the absence of lead (Pb) in Cs_2_AgBiBr_6_ adds significant value, making it an attractive candidate for environmentally friendly photodetectors.

## 4. Conclusions

The investigation of CsPbBr_3_ and Cs_2_AgBiBr_6_ composite thick films demonstrates their promising potential for photodetector applications. The incorporation of a polymer matrix into these perovskites improves structural integrity and enhances charge transfer while maintaining their fundamental optical properties. CsPbBr_3_ exhibits superior photocurrent responses due to its higher carrier mobility, making it suitable for general photodetector use. In contrast, Cs_2_AgBiBr_6_, despite its higher resistivity, shows potential for applications requiring high photon energy detection, such as X-ray imaging. The findings underscore the versatility of perovskite-based composites in optoelectronic devices and emphasize the benefits of lead-free materials for environmentally sustainable technologies. Future research should focus on optimizing the performance and scalability of these materials to meet practical device requirements.

## Data Availability

The raw data supporting the conclusions of this article will be made available by the authors on request.

## Supplementary Materials

The following supporting information can be downloaded at: https://www.mdpi.com/article/10.3390/ma17205123/s1, Figure S1. UV-Vis spectroscopy of PBM (Poly (butyl methacrylate)); Figure S2. Equivalent circuit for dark and illuminated condition. *R_s_*: Series resistance, *R_p_*: Transfer resistance, *R_ct_*: Charge transfer resistance, *R_tr_*: Transfer resistance, *C_dl_*: Interfacial capacitance, *C_g_*: Geometric capacitance, *L_s_*: Series inductance, Warburg opens; Figure S3. Histogram of grain size distribution.
