# Supplementary material for: CsPbBr3 and Cs2AgBiBr6 Composite Thick Films with Potential Photodetector Applications
Source: Materials (Basel). 2024 Oct 21;17(20):5123. doi: 10.3390/ma17205123 (PMC11509220; doi:10.3390/ma17205123)
Supplement: Supplementary file 1 [file materials-17-05123-s001.zip › materials-3237815-supplementary.pdf]

$CsPbBr_3$ and $Cs_2AgBiBr_6$ Composite Thick Films with Potential Photodetector Applications

Merida Sotelo-Lerma [1,2], Leunam Fernandez-Izquierdo [2], Martin A. Ruiz-Molina [1,2], Igor Borges-Doren [1,2] Ross Haroldson [2] and Manuel Quevedo-Lopez [2,*]

[1] Department of Research in Polymers and Materials, Universidad de Sonora, Blvd. Luis Encinas y Rosales S/N, Hermosillo, Sonora, 83000, Mexico.
[2] Materials Science and Engineering Department, University of Texas at Dallas, 2601 North Floyd Road, RL 10, Richardson, TX, 75080, USA.
* Correspondence: mquevedo@utdallas.edu

Supplementary

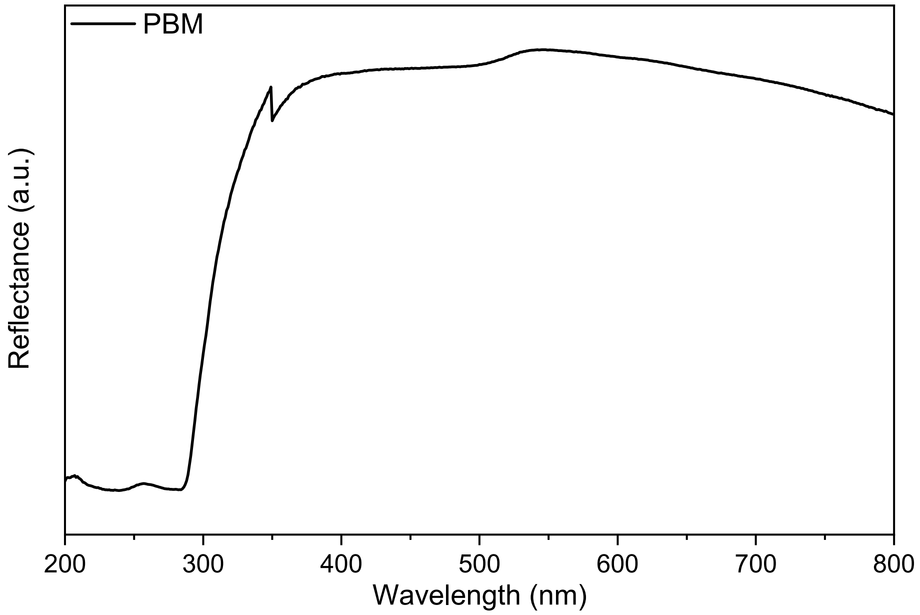

Figure S1. UV-Vis spectroscopy of PBM (Poly (butyl methacrylate))

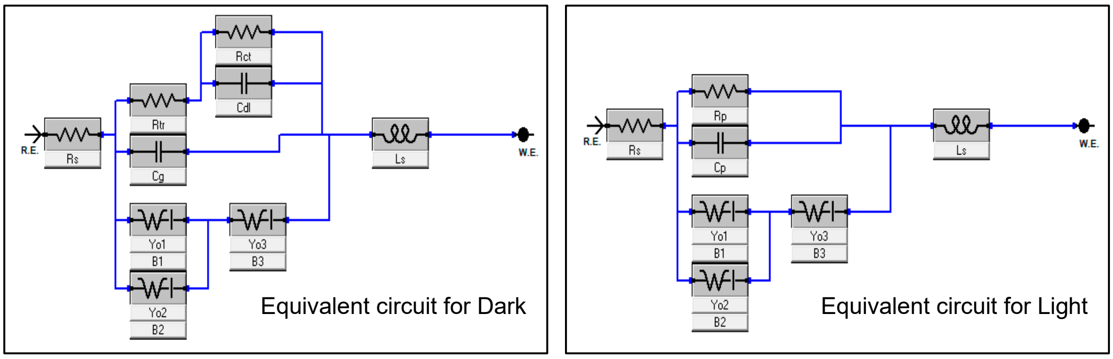

Figure S2. Equivalent circuit for dark and illuminated condition. R_s: Series resistance, R_p: Transfer resistance, R_{ct}: Charge transfer resistance, R_{tr}: Transfer resistance, C_{dl}: Interfacial capacitance, C_g: Geometric capacitance, L_s: Series inductance, Warburg opens.

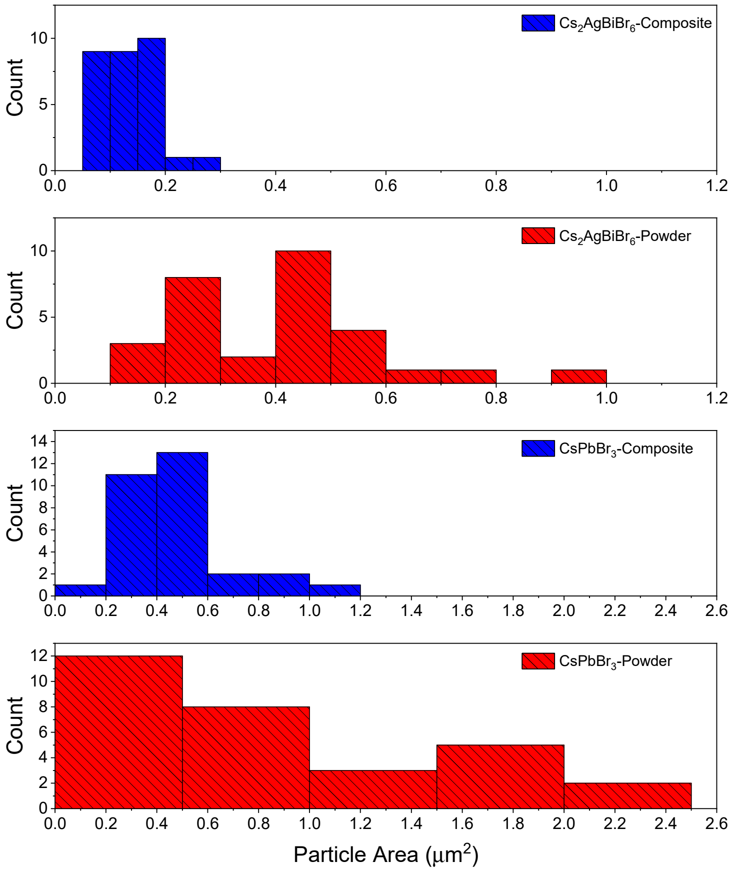

Figure S3. Histogram of grain size distribution
